# Coding and non-coding RNA interactions reveal immune-related pathways in peripheral blood mononuclear cells derived from patients with proliferative vitreoretinopathy

**DOI:** 10.1186/s12920-021-00875-5

**Published:** 2021-01-28

**Authors:** Yao Ni, Fangyuan Liu, Xiao Hu, Yingyan Qin, Zhaotian Zhang

**Affiliations:** State Key Laboratory of Ophthalmology, Zhongshan Ophthalmic Center, Sun Yat-Sen University, 54S Xianlie Road, Guangzhou, 510060 China

**Keywords:** Proliferative vitreoretinopathy, Gene expression profile, Long non-coding RNAs, Immune-related pathway

## Abstract

**Background:**

Peripheral immune response has been revealed to play a critical role in proliferative vitreoretinopathy (PVR). However, the reliable immune-related factors that are acting as prognostic indicators or therapeutic targets for PVR remain to explore further.

**Methods:**

In the current study, we applied whole-transcriptome sequencing to profile peripheral blood mononuclear cells from PVR patients and also analyzed lncRNA-mRNA interactions in peripheral immune cells to explore the pathways that might mediate immunopathology and resultant retinal damage in PVR. Gene ontology (GO), Kyoto Encyclopedia of Genes and Genomes (KEGG) pathway enrichment analyses and Ingenuity Pathway Analysis (IPA) were employed to classify the function of these differentially expressed genes.

**Results:**

Compared to the controls, there were 319 genes upregulated, and 191 genes downregulated in PVR patients. GO, and KEGG enrichment analyses as well as IPA showed that these upregulated genes were significantly enriched in immune-related and infection-relate terms. Immune-related gene *NFKBIA*, *CXCL2*, and *CXCL8* were detected as hub-genes in the co-expression network, while lncRNAs such as *AC007032.1*, *AC037198.2*, *AL929472.2*, and *SLED1* were highly co-expressed with them. lncRNA-mRNA interactions analysis also showed that putative targeted genes of these differentially expressed lncRNAs were also significantly enriched in immune-related or infection-relate pathways.

**Conclusion:**

Our study highlights the transformation of immune-related genes/pathways in PVR by comparing controls, and validates several critical genes and lncRNAs, which are serving as potential diagnostic markers for PVR patients.

## Background

Proliferative vitreoretinopathy (PVR) is a critically blinding complication that occurs during rhegmatogenous retinal detachment (RRD), before or after surgery. It is caused by the proliferation of glial cells or RPE cells to form a fibrous membrane at the neural retinal surface or even in the retina [1]. Immune cells involved in the pathogenesis of PVR, such as monocyte/macrophage infiltration and activity contributed to the progress of PVR [2]. And, some inflammatory-related genes are identified that can predict the susceptibility of PVR in populations [3]. Recently, the interaction of long non-coding RNAs (lncRNAs) and protein-coding RNAs (mRNAs) has been revealed playing a significant role in diseases related to inflammation and immunity [4, 5]. However, despite some lncRNAs, such as *MALAT1* that had been unveiled by some publications [6, 7], there are no reliable lncRNAs currently implicating in clinical practice acting as prognostic indicators or therapeutic targets for PVR patients. Thus, subsequent studies to identify more critical lncRNAs associated with PVR are warranted.

In the present study, we hypothesized that transcriptomes of peripheral immune cells in PVR might be altered, which are the potential cause for the initiation or progression of PVR. Peripheral blood samples were taken from patients undergoing standard three-port pars plana vitrectomy for indications of PVR secondary to RRD and the entire transcriptome sequencing was performed. Patients with idiopathic epiretinal membrane (iERM) underwent pars plana vitrectomy were used as controls [8,9]. Patients were excluded from analysis if they were with systemic diseases (e.g., diabetes, immunological diseases, infections, etc.) that could influence systemic inflammation. With the sequencing data, we use lncRNA associated genes to applied Gene Ontology (GO) analysis, Kyoto Encyclopedia of Genes and Genomes (KEGG) analysis and Ingenuity Pathway Analysis (IPA), as well as gene-lncRNA co-expression analysis to see the differences in genes and transcripts level between PVR patients and controls. We also validated some selected differentially expressed lncRNAs by quantitative real-time polymerase chain reaction (qPCR) assay. The results of the current study provide novel insights into PVR pathogenesis and treatment therapeutic targets.

## Methods

### Ethics statement and clinical sample collection

This study was approved by the ethics committees of the Zhongshan Ophthalmic Center. The peripheral blood samples were taken in accordance with the Declaration of Helsinki and written consent was obtained from all participants. Patients diagnosed as primary rhegmatogenous retinal detachment (RRD) with serious PVR (≥ Grade C) [10], and were scheduled to have pars plana vitrectomy (PPV) from October 2018 to February 2019 were included (PVR group). Patients diagnosed as idiopathic epiretinal membrane (iERM) and scheduled to have PPV during the same period were included as negative controls (iERM group). The blood samples were collected from patients before surgery.

### RNA extraction and cDNA library construction

Using Ficoll-Paque™ PREMIUM Media (GE Healthcare Life Sciences, Massachusetts, America) and SepMate™-50 (STEMCELL Technologies, Vancouver, Canada), approximately 5 mL of anticoagulated peripheral blood was centrifuged at 500 g for 30 min, and the thin white layer was collected as PBMC. RNeasy Mini Kit (Qiagen Corporation, Hilden, Germany) was used to extract the total RNA from PBMC. After using Agilent 4200 Bioanalyzer (Agilent Technologies, Santa Clara, CA, USA) to determine the RNA integrity number and quantity, the rRNA was removed by Epicentre Ribo-zero rRNA Removal Kit (Epicentre, Madison, WI, USA). Subsequently, the DNA was cleaned by DNase and then captured and purified by magnet beads (Vazyme, Nanjing, China). The purified RNA was interrupted into short fragments by adding fragmentation buffer, then the first-strand cDNA was synthesized and double-strand cDNA was obtained thereafter with VAHTS Universal V6 RNA-seq Library Prep Kit for Illumina (Vazyme, Nanjing, China). Adapters were then connected to the cDNAs and Agilent 4200 Bioanalyzer, as well as qPCR, were used to verify the fragment size and amplify the templates. The constructed library was then loaded on the Illumina HiSeq X Ten system for sequencing.

### Sequence analysis and functional annotation

We used FastQC first to check the quality of raw reads and then processed with Cutadapt software to generate clean reads by trim adaptor sequences and removed the low-quality sequences [11]. HISAT2 was used to align the clean reads to the hg38 human reference genome [12]. Mapped reads used the StringTie software and Ensembl GTF file(release 90) to obtain gene expression profiles [13–15], and then the differential expression analysis was executed via the DESeq2 package on R programmer [16]. |log2Fold Change|≥ 0.585 (Fold change ≥ 1.5 or Fold change ≤ 0.6667) and *P* value < 0.05 were decided upon as the significance of differentially expressed genes. GO and KEGG enrichment analyses were then performed using the Clusterprofiler R package.17 And then use WCGNA package on R to calculate the person coefficient and Construction of hierarchical clustering trees by correlation coefficients between genes. Then export to Cytoscape software to draw the network plot. For further functional analysis, differentially expressed genes and transcripts between PVR and iERM patients, including gene symbols and expression values were uploaded into IPA software (Qiagen, Germany). The canonical pathways, diseases and biofunctions as well as gene networks analysis were processed.

### QPCR validation

Total RNA was isolated as mentioned above, and the cDNA was then synthesized with HiScript II Q Select RT SuperMix for qPCR (Vazyme, Nanjing, China). Roche lightcycler 96 was then used to perform qPCR. Beta-actin was used as the internal control. The melting curve was used to confirm reaction specificity and relative expression was calculated by the 2^−ΔΔCt^ method. Primer sequences and product length were listed in Table 1.

**Table 1:** Primers of the validated lncRNAs

Gene	Primer Sequence (5′ − 3′)	Product length
AC037198.1	F: CCTCATACTCGCGCATTCTT	139 bp
	R: GCCTTCCCACAGTGTATGCT	
ZNF433-AS1	F: CCGGAATATCTGGAAGCTGA	111 bp
	R: GTCTCAATGGCACCCAGATT	
Beta-actin	F: CTCTTCCAGCCTTCCTTCCT	116 bp
	R: AGCACTGTGTTGGCGTACAG	

### Statistical analyses

GraphPad Prism 7.0 (GraphPad company, San Diego, USA) was used to compare the lncRNA expression differences obtained by qPCR assay referring to the Mann–Whitney U test between PVR and iERM groups. A *P* value ≤ 0.05 was considered statistically significant.

## Results

### Characteristics of the subjects

There were six males and six females in the PVR group, and eight males and five females in the iERM group. The average age of the PVR group was 53.3 ± 10.6 years old, and that of the iERM group was 60.0 ± 9.7 years old. The differences in age and gender between PVR and iERM groups were not statistically different (*P* > 0.05).

### Identification of differentially expressed genes and transcripts

**Fig. 1:**
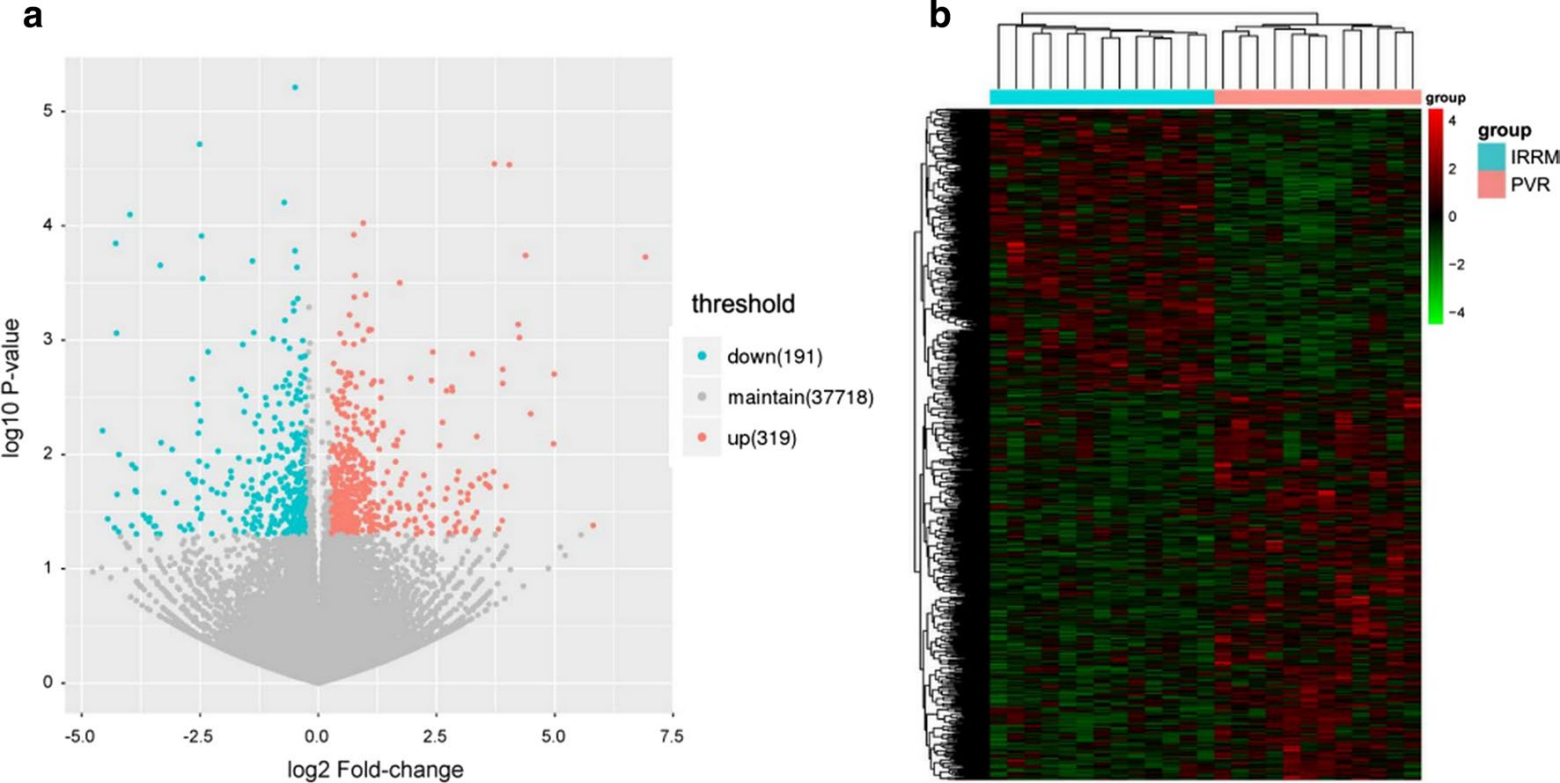
Differential gene expression. **a** Volcano plot assessment of gene expression between iERM and PVR patients (blue dots indicate downregulated genes and red dots indicate upregulated genes), and **b** Heatmap showing unsupervised cluster analysis of differentially expressed gene between iERM (Blue) and PVR (Red) patients. iERM, idiopathic epiretinal membrane; PVR, proliferative vitreoretinopathy

Using 1.5-fold expression difference as a cutoff, 510 genes were found differentially expressed between PVR patients and iERM patients, among which 319 were upregulated and 191 were downregulated (Fig. 1a). As expected, these two kinds of patients could be clustered into separated groups using the differentially expressed genes (DEGs) (Fig. 1b), highlighting the apparent genes difference between PVR and iERM. Differentially expressed transcripts between PVR and iERM was also analyzed in this study.

Besides, for the 5,138 differentially expressed transcripts (DETs) (Additional file 3: Figure S1), 64.38% of them were protein-coding RNAs, whereas 19.26% were lncRNAs (Additional file 3: Figure S2A). The length of lncRNA varies from 1,000 to larger than 10,000 (Additional file 3: Figure S2B). We also performed an unsupervised cluster analysis with the DETs. Similar to the gene clustering analysis, samples were significantly separated in accordance with two patient groups.

### Gene ontology analysis and KEGG analysis

**Fig. 2:**
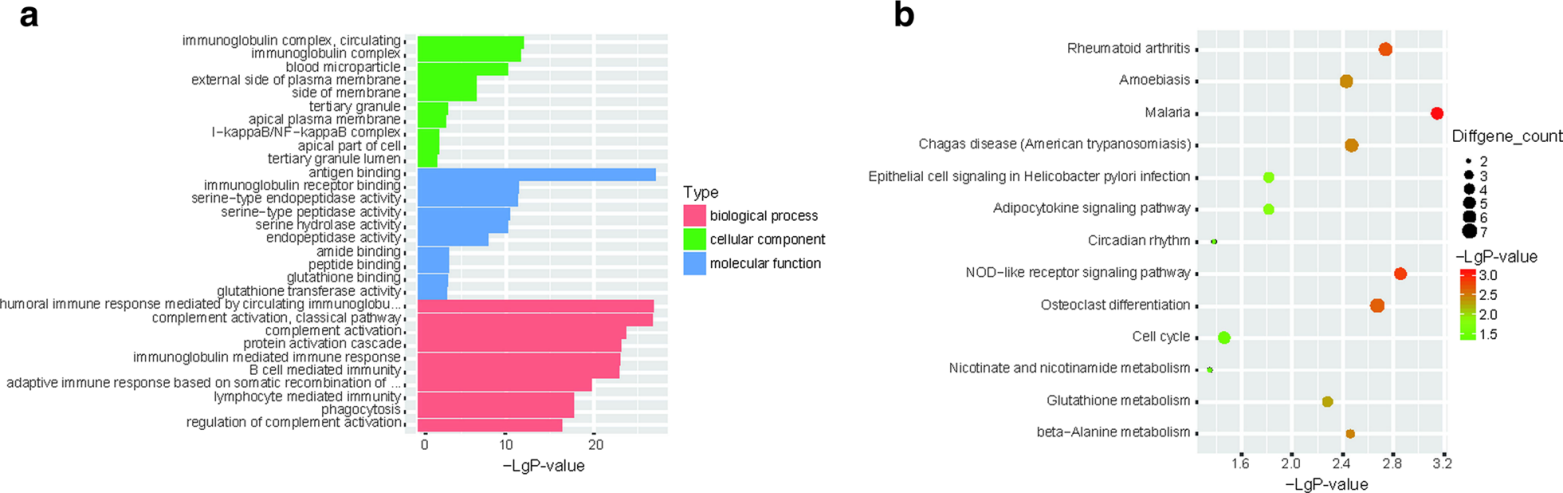
Pathway analysis of upregulated genes between iERM and PVR patients. **a** GO analysis data and **b** KEGG analysis data. GO, Gene Ontology; KEGG, Kyoto Encyclopedia of Genes and Genomes

To explore the biology underlying the deferentially expressed gene further, we performed an overrepresentation analysis of GO terms and KEGG pathways using the R program with the ClusterProfiler package. It is very interesting to note that most of the upregulated genes were enriched in immune system-related terms, including immunoglobulin, complement, and immune response (Fig. 2a); while the downregulated GO terms were irrelevant to the immune system (Additional file 3: Figure S3A).

Upregulated genes of PVR patients compared with iERM patients were enriched in many pathways from KEGG (Fig. 2B). To our surprise, many infectious pathways, including malaria, legionellosis, and Chagas disease, were involved with high significance. Besides, pathways affecting the immune system, including IL-17 signaling, TNF signaling, as well as rheumatoid arthritis, were enriched. Much different from the upregulated genes, the downregulated genes were poorly enriched in KEGG pathway enrichment (Additional file 3: Figure S3B).

### Enriched pathway, diseases, biofunctions and interaction network by IPA analysis

**Fig. 3:**
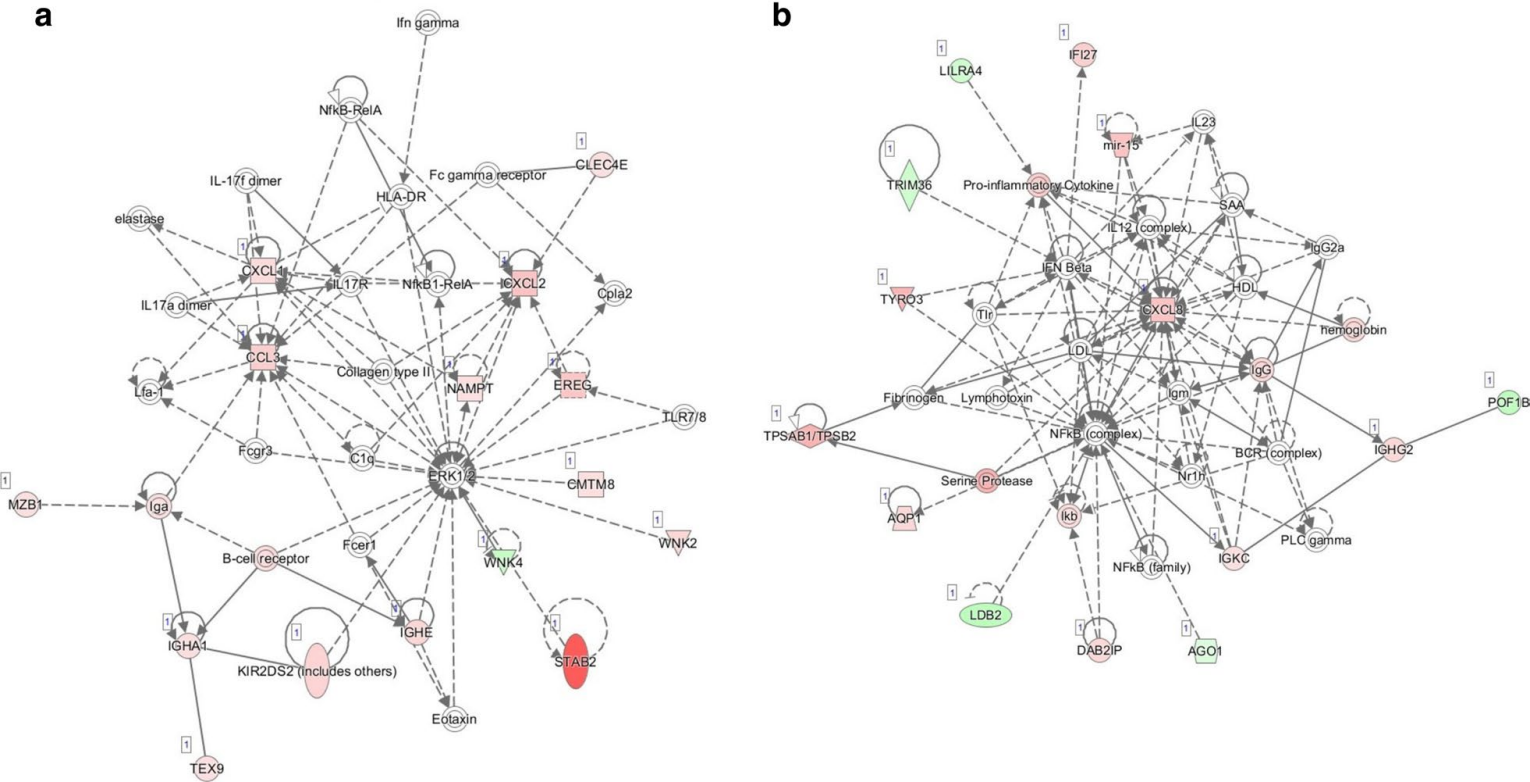
Ingenuity Pathway Analysis (IPA)-identified immune related gene networks with score > 20. **a** Cell-To-Cell Signaling and Interaction, Cellular Movement, Immune Cell Trafficking; **b** Gastrointestinal Disease, Inflammatory Disease, Inflammatory Response

We used IPA to analysis the significant changed 751 lncRNA associated genes (*P* < 0.05 and fold-change > 1.5) between patient PVR and iERM patients. With IPA, we revealed these changed genes are closely related to 26 canonical pathways. The most significant of those are shown in Table 2. 5 out of top ten pathways are related to innate and adaptive immune cells especially Th17 cells and its cytokines IL-17A and IL-17F. With IPA, we also found similar change as we revealed in GO and KEGG analysis: Pathways related to immune reaction were also enriched. For the top four scored networks (Additional file 4: Table S1), 2 of them were immune function related. These two networks are shown in Fig. 3: Cell-To-Cell Signaling and Interaction, Cellular Movement, Immune Cell Trafficking (score 23, Fig. 3a); and Gastrointestinal Disease, Inflammatory Disease, Inflammatory Response (score 21, Fig. 3b).

**Table 2:** Top significantly enriched canonical pathways of protein coding RNAs in PVR patients

Ingenuity canonical pathways	-log(*p* value)	Ratio	Molecules
Communication between innate and adaptive immune cells	3.53	0.0625	CCL3, CXCL8, IGHA1, IGHE, IGHG2, IL1B
Differential regulation of cytokine production in macrophages and T helper cells by IL-17A and IL-17F	3.22	0.167	CCL3, CXCL1, IL1B
Differential regulation of cytokine production in intestinal epithelial cells by IL-17A and IL-17F	2.9	0.13	CCL3, CXCL1, IL1B
Role of IL-17A in arthritis	2.75	0.0727	CXCL1, CXCL2, CXCL8, NFKBIA
Airway pathology in chronic obstructive pulmonary disease	2.63	0.25	CXCL2, CXCL8
Glutathione-mediated detoxification	2.48	0.0938	GSTM1, GSTM4, HPGDS
TREM1 signaling	2.26	0.0533	CCL3, CXCL2, CXCL8, IL1B
Role of IL-17A in psoriasis	2.2	0.154	CXCL1, CXCL8
Atherosclerosis signaling	2.17	0.0397	COL1A1, CXCL8, IL1B, PLAAT2, TPSAB1/TPSB2
Role of IL-17F in allergic inflammatory airway diseases	2.15	0.0714	CXCL1, CXCL8, IL1B

### mRNA and lncRNA co-expression analysis

**Fig. 4:**
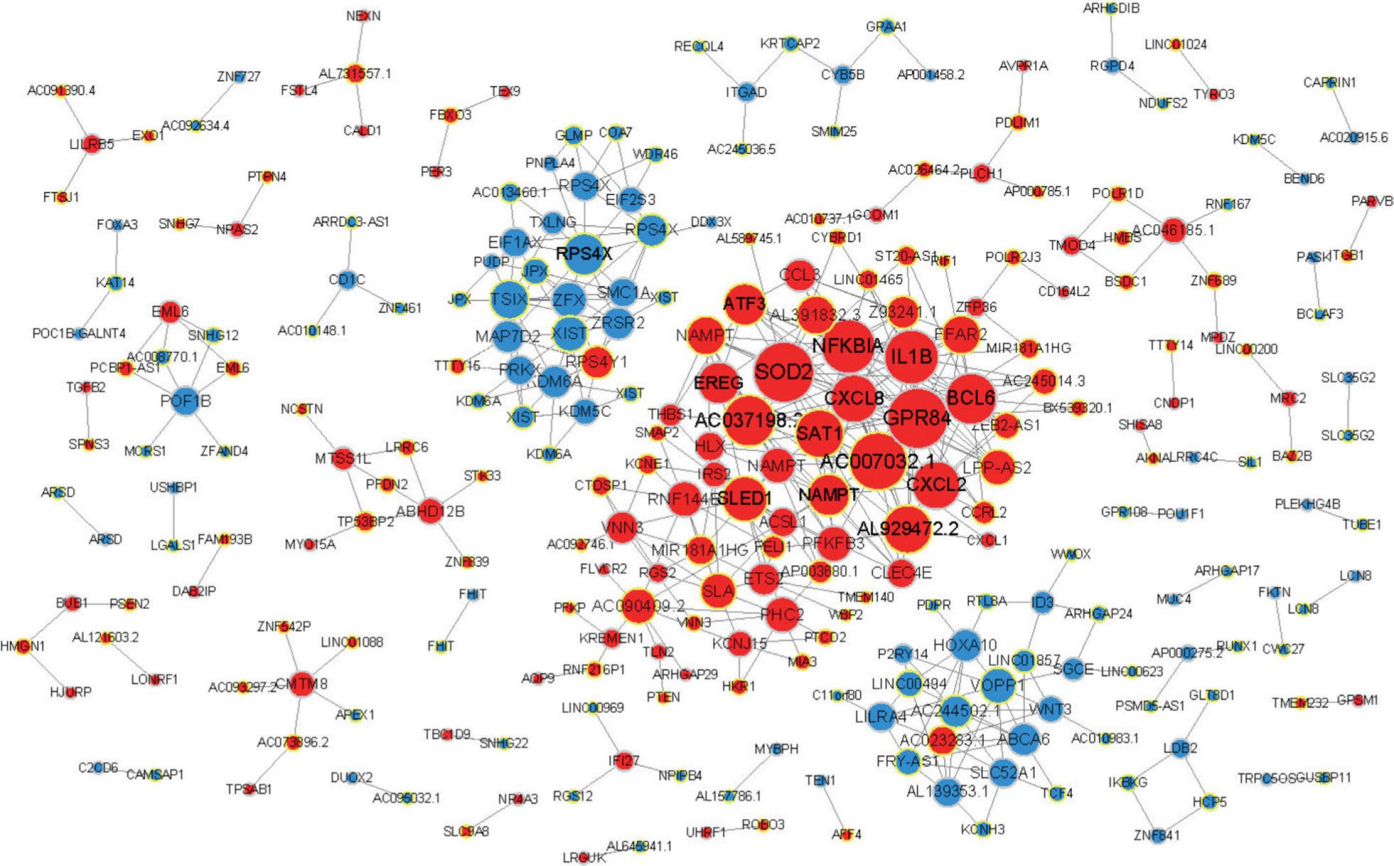
Co-expression network of gene-lncRNA network. Red dots indicate upregulated gene/lncRNA and blue dots indicate downregulated gene/lncRNA; solid line represents positive relation, and the dotted line represents negative relation

As LncRNAs are also important participants in disease process. Based on the correlation of the significantly regulated protein-coding mRNA with lncRNA, we constructed co-expression networks to analyze their interaction and to find out the potential therapeutic target. Pearson correlation coefficient was calculated for each pair and significantly correlated RNA pairs were chosen (r > 0.8, *P* < 0.05 as the threshold), 245 pairs of mRNA-lncRNA pairs were obtained. As shown in Fig. 4, immune-related gene *NFKBIA*, and chemokines *CXCL2* and *CXCL8* were of high hubness in the co-expression network, highlighting their important biological role in the difference between PVR and iERM. Besides, we found several lncRNAs were highly co-expressed with these genes, including *AC007032.1*, *AC037198.2*, *AL929472.2*, *SLED1*, etc., indicating the key regulation values of them.

**Fig. 5:**
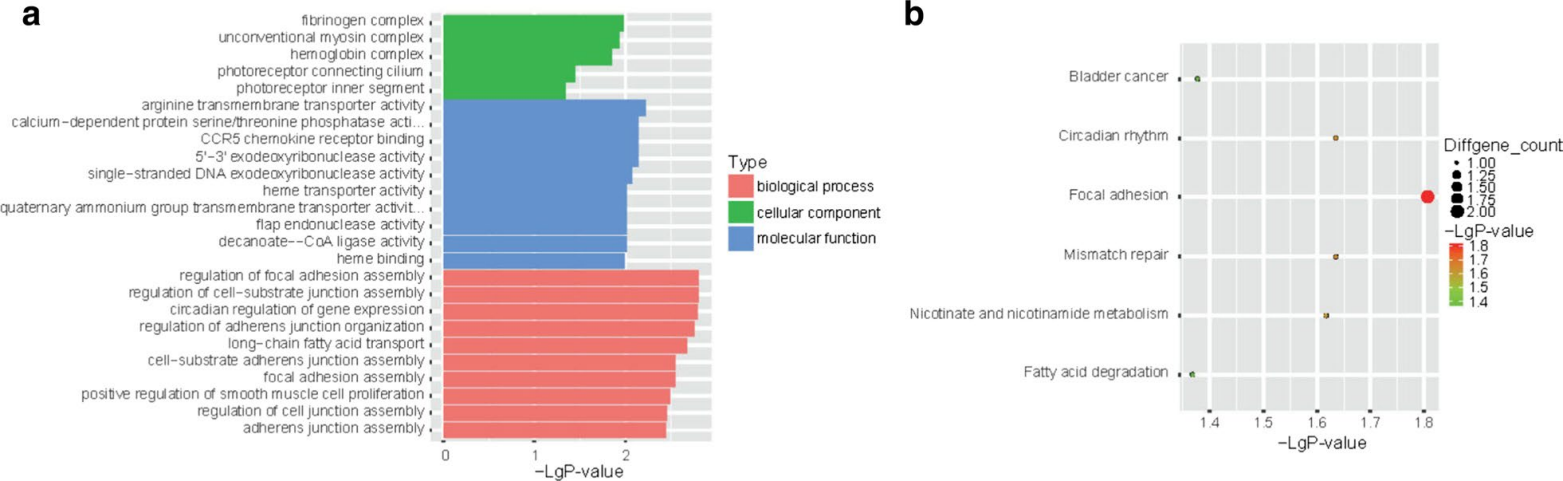
Pathway analysis of upregulated lncRNAs between iERM and PVR patients. **a** GO analysis data and **b** KEGG analysis data. GO, Gene Ontology; KEGG, Kyoto Encyclopedia of Genes and Genomes

We searched further to look for the lncRNA-mRNA interactions, inspecting the chromosome location of regulated lncRNAs to see if they had up- or down-stream 10 kb or overlapping mRNAs. As a result, 630 lncRNAs that had nearby DEGs were found, including 290 upregulated and 340 downregulated lncRNAs. GO, and KEGG analyses and also IPA were conducted as above (Fig. 5 and Table 3). Like that of DETs GO analysis, the upregulated lncRNAs had more enriched GO terms compared to the downregulated lncRNAs though few of which was immune related (Fig. 5a and Additional file 3: Figure S4A). For the KEGG analysis, fewer pathways were enriched (Fig. 5b and Additional file 3: Figure S4B). results above might due to many functions of lncRNA that are still unclear. However, in IPA analysis, though none of the top ten enriched pathways were immune related pathways, we found Virus Entry via Endocytic Pathways were enriched. Further, the top 2 scored networks are relate to Ophthalmic Disease or Immunological Disease respectively (Additional file 5: Table S2).

**Table 3:** Top significantly enriched canonical pathways of LncRNAs in PVR patients

Ingenuity canonical pathways	−log(*p* value)	Ratio	Molecules
D-myo-inositol-5-phosphate metabolism	2.85	0.0641	DOT1L, INPP5B, MDP1, NUDT5, PLCG1, PPIP5K1, PPP2R5A, PTPN6, PTPRM, SET
Superpathway of inositol phosphate compounds	2.12	0.0505	DOT1L, INPP5B, MDP1, NUDT5, PLCG1, PPIP5K1, PPP2R5A, PTPN6, PTPRM, SET
Endocannabinoid cancer inhibition pathway	2.04	0.0559	ATF3, CREB1, GNB1L, LEF1, MAP2K4, PRKACA, SPTLC1, TCF4
Heme biosynthesis II	1.87	0.222	HMBS, UROD
Folate transformations I	1.87	0.222	MTHFD2, MTHFR
Salvage pathways of pyrimidine ribonucleotides	1.84	0.0619	CSNK1D, GRK4, MAP2K4, MAPK6, PRKCH, UCKL1
Protein kinase a signaling	1.76	0.0377	ADD1, ADD3, APEX1, CHP1, CREB1, FLNA, GNB1L, LEF1, PLCG1, PRKACA, PRKCH, PTPN4, PTPN6, PTPRM, TCF4
Amyloid processing	1.73	0.08	CAPN2, CSNK1D, PRKACA, PSEN2
Androgen signaling	1.66	0.0515	GNA12, GNB1L, KAT7, NCOA1, POLR2J2/POLR2J3, PRKACA, PRKCH
Virus entry via endocytic pathways	1.65	0.0561	AP2A1, FLNA, ITGAL, ITGB1, PLCG1, PRKCH

### Validation of differentially expressed lncRNAs

**Fig. 6:**
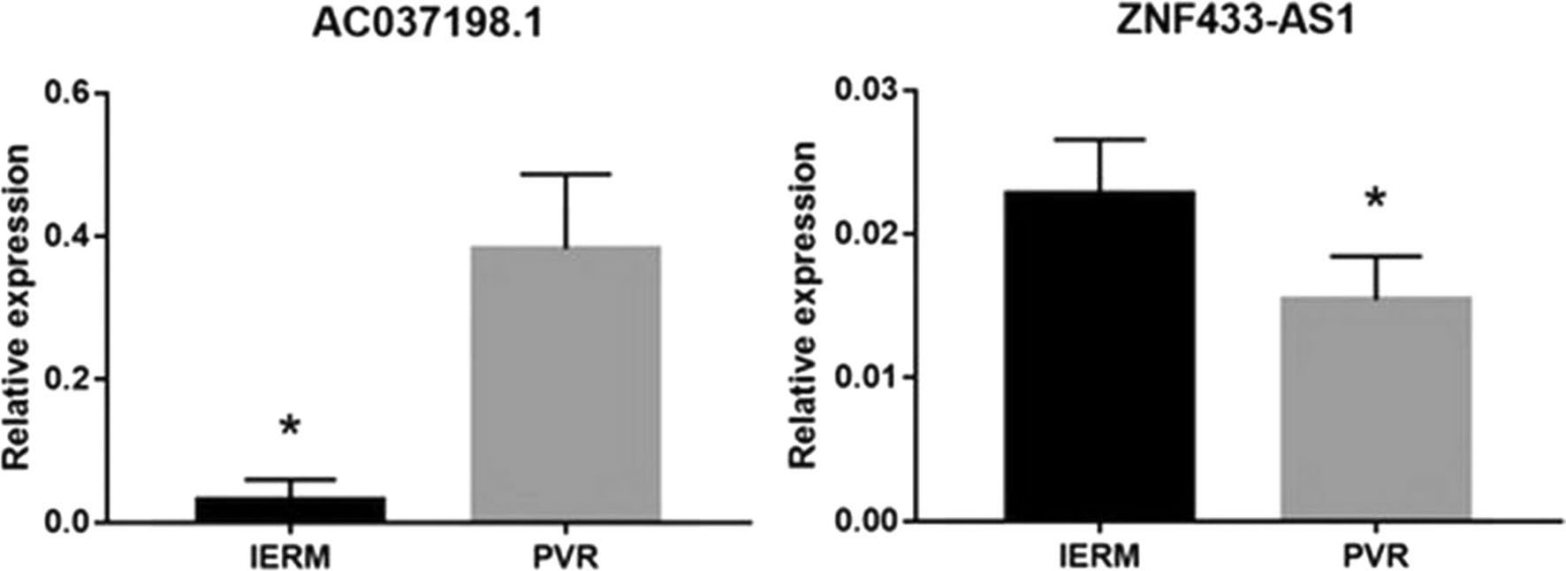
Validation of two selected lncRNAs. The expression level was normalized to the housekeeping gene beta-actin (*P < .05)

We used qPCR assay to validate the deferentially expressed lncRNAs, while *AC037198.2*, and *ZNF433-AS1* were selected based on differential expression and co-expression analyses. The differential expressed gene and transcripts are listed in “Additional file 1” and “Additional file 2”, respectively. As shown in Fig. 6, these lncRNAs were significantly different among PVR and iERM patients. The *AC037198.2* expressed 14.5 times higher in PVR group while *ZNF433-AS1* was expressed 30.7% less in PVR patients when compared to iERM patients.

## Discussion

PVR is still the leading cause of vitreoretinal surgery failure, mainly through retina re-detachment and even intraretinal fibrosis. The incidence of PVR is estimated to be 5% to 10% [18]. However, as the mechanisms of PVR are still not very clear, it is difficult to predict or treat the condition despite the many efforts that are still being made. In the present study, our results identified differentially expressed mRNA and lncRNA in PBMCs of PVR patients and revealed that gene expression profile and molecular signature of PVR patients.

The proliferation of cells, mainly RPE and glial cells, is the essential point of PVR development. Clinicians have tried for more than four decades to inhibit the proliferation of cells in the vitreous to prevent PVR but have not had impressive progress [18]. This situation has raised the question of whether there is anything abnormal out of the eye in this disease. The answer to this might come from the immune system.

In the review by Pastor et al., the authors proposed that ischemia, blood-retina barrier breakdown, and inflammation lead to the final PVR based on the collaborative genetic study named “Retina 4 Project”, which found 30 inflammatory-related genes were responsible for PVR [4]. Further, a single nucleotide polymorphism (SNP) analysis in peripheral blood from PVR patients shown that TNF locus which encompasses the gene of TNFα contributes to the development of PVR [19]. It is suggested that PVR might not only be a “local inflammatory condition’’, but also could be affected by system regulation.

Compare with these studies, with RNA from peripheral blood mononuclear cells and with GO, KEGG and IPA analysis, we revealed that the immune related pathways or components are closely related to PVR change. We found immunoglobulin and its receptors as well as antigen binding were all up regulated in PBMC from PVR patient. Further, RNAs related to biological process of adaptive immune response and lymphocytes activation were also up regulated indicating microbial infection might play a role in the disease process. In IPA analysis, we found in enriched canonical pathways, CCL1, CCL3, CXCL8 and especially Th17 cells and its related cytokines IL-17A and IL-17F were emphasized. In previous publication, Th17 and its cytokines are inflammatory mediator to RPE cells [20], and RPE cells in inflammatory condition were thought to be a key point to PVR formation [18].

Infection had long been considered as a trigger to immune disease. In the KEGG analysis, to our surprise, rather than immune pathways, infectious related pathways related to amoebiasis, malaria or chagas disease were enriched. As in uveitis, Forrester et al. agreed that infection may directly or indirectly related to noninfectious uveitis in the eye [21]. In this consideration, remind us that infection might be a potential cause of PVR.

LncRNAs are widely expressed in monocytes, macrophages, neutrophils and implicated in the process of inflammation and immunity. Various molecular functions have been ascribed to lncRNAs, including gene regulation in cis, regulation of mRNA stability, and modulation of protein function. In PVR patients, Zhou et al. not only demonstrated that the expression of MALAT1 was significantly upregulated in the proliferating membrane, also found MALAT1 was significantly up-regulated in the peripheral blood [6]. MALAT1 can inhibit the DNA binding activity of NF- κB, reduce the production of inflammatory cytokines, and down-regulate the autoimmune inflammatory response. The knockdown of MALAT1 can increase lipopolysaccharide (LPS)-induced expression of TNFα and IL-6 [22]. However, to our surprise, we did not find MALAT1 were upregulated in PBMC in PVR patient.

In our research, by analysis the mRNA and lncRNA co-expression in PBMC, we found immune-related gene *NFKBIA*, and chemokines *CXCL2* and *CXCL8* and their associate LncRNA *AC007032.1*, *AC037198.2*, *AL929472.2*, *SLED1* were highly associated with PVR. *AC007032.1* is associated with immunomodulatory cytokine Nampt [23], while *SLED1* was found up regulated in peripheral blood cells of systemic lupus erythematosus patients [24]. Additionally, within IPA analysis, virus infection relate pathways and immune relate networks were highlighted in PVR patients.

Further, the most obvious changed LncRNA *AC037198.2* and *ZNF433-AS1* were selected to verify and were proved their actual change in PBMC from PVR patients. LncRNA AC037198.2 is associate with THBS1(thrombospondin 1) gene, which encoded a secreted protein to mediate cell-to-cell and cell-to-matrix interactions. As for *ZNF433-AS1*, this LncRNA can suppress ZNF433, which belongs to transcriptional factors with the zinc finger motif, and was found that play an important role in multiple sclerosis, which is an autoimmune disease^25^.

There were still some limits in our study. During the data processing, we performed differential comparison of genes and transcripts by DESeq2, but the limited sample size and relatively large individual differences in human samples made it difficult to pass the FDR test. Alternatively, in order to furtherly verify the authenticity of the p-value screen for significantly different genes, we performed qPCR quantitative validation on the same patient samples, and the results were consistent with the sequencing results. Therefore, we continued to use the p-value screened differential genes for subsequent analysis.

## Conclusions

In summary, we provide a landscape of differential expression profile of mRNAs and lncRNAs between PVR and controls and construct an mRNA-lncRNA co-expression network based on the DETs. Pathway enrichment analyses offer novel insights into the pathogenesis of PVR, indicating that PVR might be related with abnormal immune system or previous infection. More importantly, some deferentially expressed lncRNAs, like LncRNA-*AC037198.2* and *ZNF433-AS1*were appeared in our study, which might be potential molecular signatures for PVR. These results will provide hence our understanding of this disease and provide novel therapeutic targets for PVR patients.

## Supplementary Information

**Additional file 1**. The differential expressed gene.

**Additional file 2**. The differential expressed transcripts.

**Additional file 3: Figure S1**. Volcano plot assessment of differentially expressed transcripts between iERM and PVR patients. **Figure S2**. **A** The distributions of the differentially expressed transcripts. **B** The lengths of differentially expressed lncRNAs. **Figure S3**. **A** Pathway analysis of downregulated genes between iERM and PVR patients-GO analysis data. **B** Pathway analysis of downregulated genes between iERM and PVR patients-KEGG analysis data.

**Additional file 4: Table S1**. Enrichend Top Diseases and Functions of differentiate expressed Protein Coding RNAs.

**Additional file 5: Table S2**. Enriched Top Diseases and Functions of differentiate expressed LncRNAs.

## Data Availability

The datasets generated during and/or analyzed during the current study have been deposited and are available from: Gene Expression Omnibus (GEO) repository with the accession ID GSE164208 (https://www.ncbi.nlm.nih.gov/geo/query/acc.cgi?acc=GSE164208); Associate data banks/repositories: Ensembl GRCh38 GTF (ftp://ftp.ensembl.org/pub/release-90/gtf/homo_sapiens/Homo_sapiens.GRCh38.90.gtf.gz); NCBI GRCh38 Genome RefSeq FASTA (https://www.ncbi.nlm.nih.gov/assembly/GCF_000001405.39; or otherwise, available from the corresponding author upon reasonable request.

## Abbreviations

PVR: Proliferative vitreoretinopathy
RRD: Rhegmatogenous retinal detachment
iERM: Idiopathic epiretinal membrane
lncRNAs: Long non-coding RNAs
PPV: Pars plana vitrectomy
GO: Gene Ontology
KEGG: Kyoto Encyclopedia of Genes and Genomes
IPA: Ingenuity Pathway Analysis
DEGs: Differentially expressed genes
DETs: Differentially expressed transcripts
SNP: Single nucleotide polymorphism
