# Supplementary material for: Coding and non-coding RNA interactions reveal immune-related pathways in peripheral blood mononuclear cells derived from patients with proliferative vitreoretinopathy
Source: BMC Med Genomics. 2021 Jan 28;14:30. doi: 10.1186/s12920-021-00875-5 (PMC7842006; doi:10.1186/s12920-021-00875-5)
Supplement: Supplementary file 3 — Additional file 3: Figure S1. Volcano plot assessment of differentially expressed transcripts between iERM and PVR patients. Figure S2. A The distributions of the differentially expressed transcripts. B The lengths of differentially expressed lncRNAs. Figure S3. A Pathway analysis of downregulated genes between iERM and PVR patients-GO analysis data. B Pathway analysis of downregulated genes between iERM and PVR patients-KEGG analysis data. [file 12920_2021_875_MOESM3_ESM.docx]

**Supplementary file and the corresponding figure legends**

**
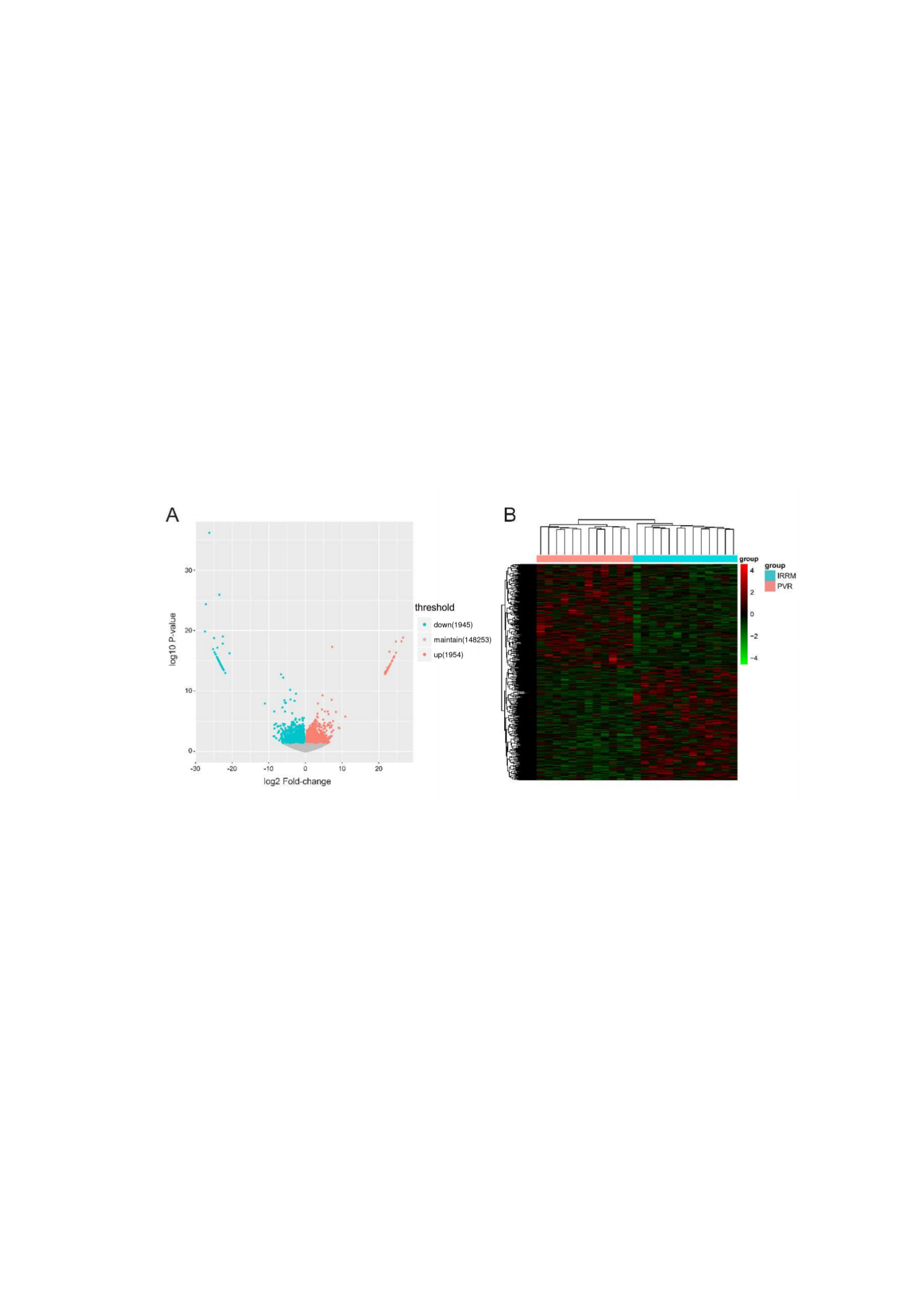
**

**Figure S1.** (A) Volcano plot assessment of transcripts between iERM and PVR patients (blue dots indicate downregulated genes and red dots indicate upregulated genes), and (B) Heatmap showing unsupervised cluster analysis of differentially expressed transcripts between iERM (Blue) and PVR (Red) patients. iERM, idiopathic epiretinal membrane; PVR, proliferative vitreoretinopathy.


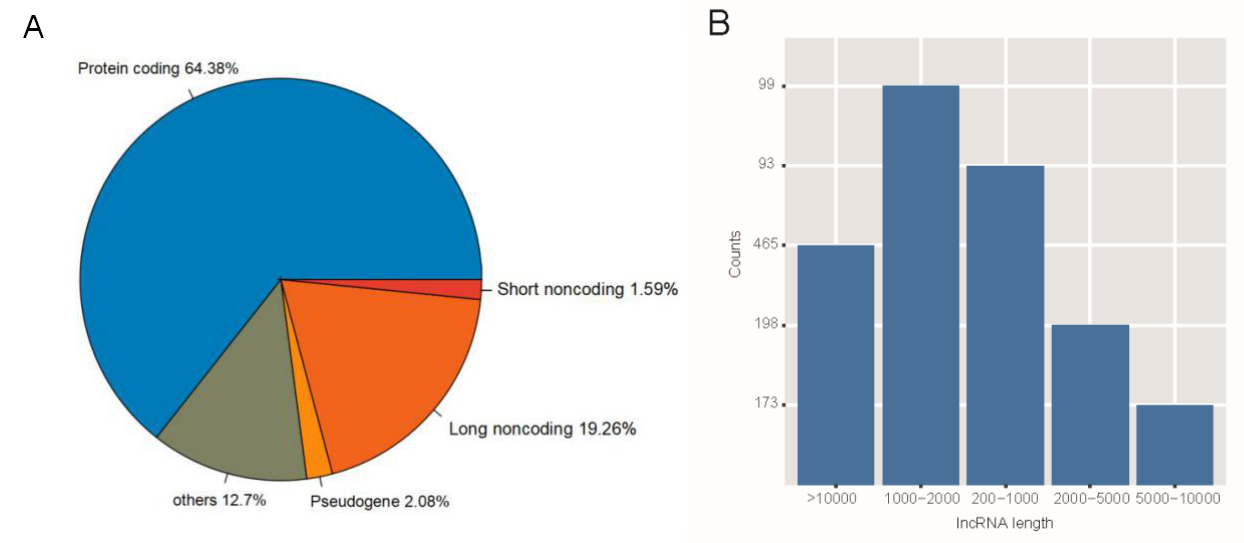


**Figure S2.** The distributions of the differentially expressed transcripts (A) and lengths of differentially expressed lncRNAs (B).


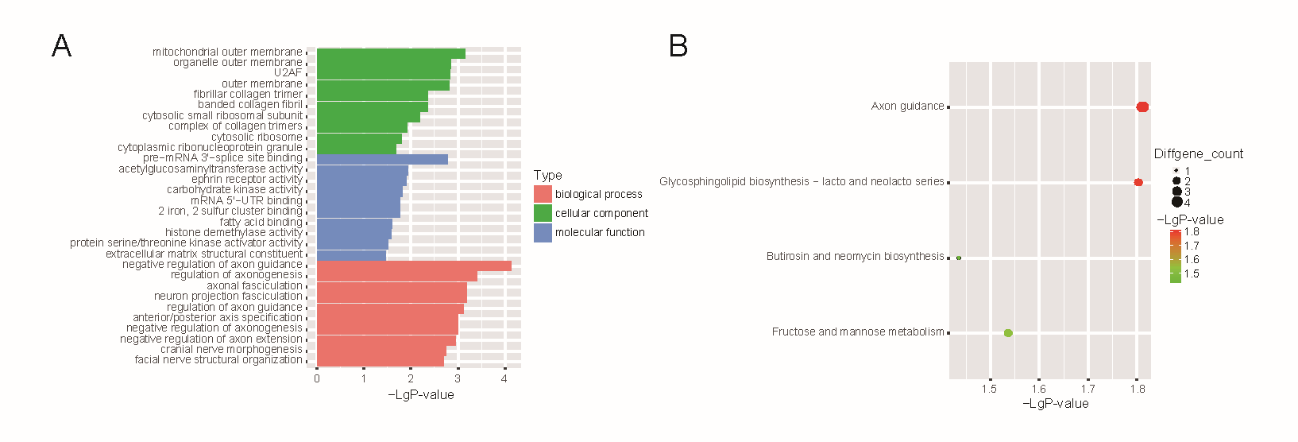


**Figure S3.** Pathway analysis of downregulated genes between iERM and PVR patients. (A) GO analysis data and (B) KEGG analysis data. GO, Gene Ontology; KEGG, Kyoto Encyclopedia of Genes and Genomes.


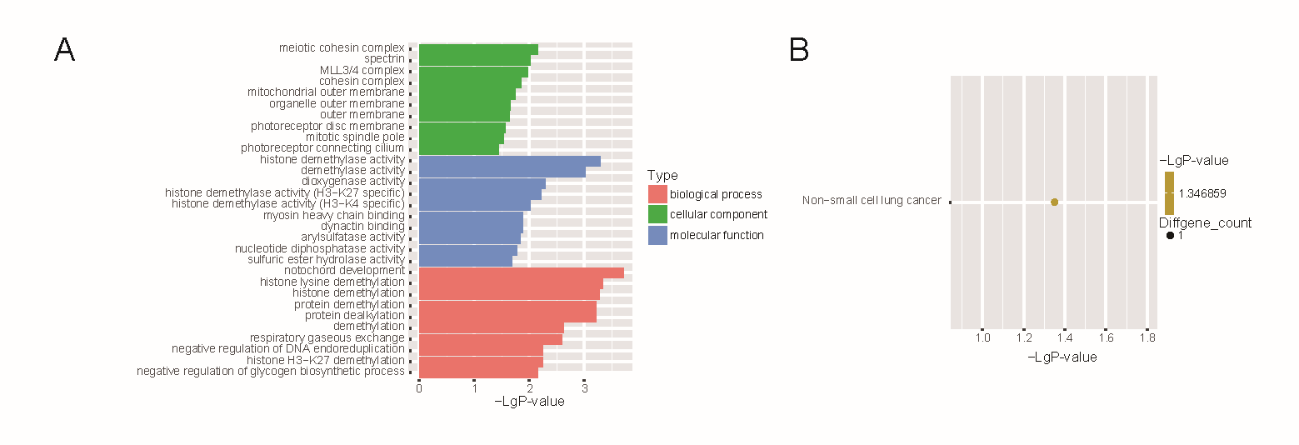


**Figure S4.** Pathway analysis of downregulated lncRNAs between iERM and PVR patients. (A) GO analysis data and (B) KEGG analysis data. GO, Gene Ontology; KEGG, Kyoto Encyclopedia of Genes and Genomes.
